# Prevalence and Psychosocial Correlations of COVID-19-Related Worries in People with Diabetes Mellitus Seeking Services from East Indian Tertiary Care Center: A Cross-Sectional Survey Results

**DOI:** 10.4103/cmi.cmi_39_21

**Published:** 2021-07-05

**Authors:** Suravi Patra, Swati Priyadarshini Acharya, Manish Taywade, Debapriya Bandyopadhyay, Binod Kumar Patro

**Affiliations:** 1 Department of Psychiatry, AIIMS, Bhubaneswar, Odisha, India; 2 Indian Council of Medical Research; 3 Department of Community Medicine and Family Medicine, AIIMS, Bhubaneswar, Odisha, India; 4 Department of Bhiochemistry, AIIMS, Bhubaneswar, Odisha, India

**Keywords:** COVID-19, psychosocial impact, type 2 diabetes mellitus, worries

## Abstract

**Context:**

Patients with diabetes are more prone to psychosocial problems which are known to adversely impact clinical outcomes of diabetes. COVID-19 is understood to further worsen the psychosocial problems of patients with diabetes.

**Aims:**

We carried out this cross-sectional telephonic survey of COVID-19-related worries in patients with diabetes mellitus to understand the prevalence and correlates of COVID-19-related worries.

**Settings and Design:**

This was a telephonic survey of patients seeking care from noncommunicable disease clinic of a tertiary care medical center.

**Subjects and Methods:**

We used a structured questionnaire to assess sociodemographic, clinical, psychological variables and COVID-19-related worries.

**Statistical Analysis Used:**

We used SPSS 20.0 for descriptive statistics keeping significance levels at 0.05. Between-group comparisons of continuous variables were made with independent *t*-test and two-way ANOVA; correlations were carried out with Pearson correlation test.

**Results:**

Two hundred and nine patients completed the telephonic survey conducted from September to November 2020. The prevalence of diabetes-related worries in our sample was 80%. Younger age (*P* < 0.001), unemployment (*P* = 0.029), and the presence of mental disorder (*P* < 0.001) were associated with higher diabetes-related worries. Poor glycemic control (0.008) and symptoms of COVID-19 (0.03) were associated with diabetes-related worries. Diabetes-related worries correlated with diabetes distress (ρ =0.441, *P* < 0.001), social isolation (ρ =0.401, *P* < 0.001), and perception of social support (ρ = −0.158, *P* < 0.001).

**Conclusions:**

A large proportion of our patients with diabetes are at high risk to experience COVID-19-related worries especially, younger people, unemployed and those with mental illness. Furthermore, the presence of diabetes distress and the perception of social isolation increase COVID-19 worries.

## INTRODUCTION

The World Health Organization declared COVID-19 a pandemic, and the government of India imposed lockdowns to contain the spread of the virus.^[1,2]^ Diabetes facilitates COVID-19 infection by enhancing the virus’s entry, having a higher affinity to virus binding and reduced virus clearance, which increases susceptibility to COVID-19 infection. High basal levels of pro-inflammatory cytokines seen in diabetes enable “cytokine storm,” worsening the prognosis of COVID-19.^[3]^

The lockdown restrictions created difficulty in diet regulation and exercise for people with diabetes.^[4]^ People with diabetes mellitus are at high risk to experience psychological distress with known adverse impact on diabetes management, self-regulation, and glycemic control. COVID-19 infection has further compounded the psychosocial difficulties of people with diabetes mellitus.^[3]^ While predictions have been made that patients with diabetes experience psychosocial issues, to our knowledge, COVID-19-specific worries in patients with diabetes mellitus have not been systematically studied or reported from the Indian population.^[5]^ We carried out this study to map COVID-19-specific worries in patients with diabetes mellitus and assess its clinical and psychosocial correlations.

## SUBJECTS AND METHODS

### Study design

We conducted this cross-sectional study from September to November 2020. Telephone numbers were retrieved from hospital records of patients seeking care from a tertiary care medical center in East India. The interviews were conducted telephonically using a semi-structured interview schedule for evaluating the psychosocial impact of COVID-19 in patients with diabetes mellitus.^[6]^

### Inclusion criteria

Patients diagnosed with diabetes mellitus and seeking care from the noncommunicable disease outpatient department of the institute were included in the study.

### Exclusion criteria

Patients not agreeing to consent were excluded from the study.

### Sample size

This was a convenience sampling; we did not carry out a formal sample size calculation as this was a descriptive study.

### Ethics

The study protocol was approved by the Institutional Ethics Committee (IEC) of our institute vide IEC no. T/IM/NF-Psychi/20/100. Verbal consent was taken from participating patients while conducting the interviews telephonically. All data were kept anonymous.

### Study instruments

We adapted a semi-structures questionnaire for evaluating the psychosocial impact of COVID-19 in diabetes.^[6]^ We adapted the questionnaire for use in our population keeping view of our sociocultural context. This questionnaire has four sections: section I is on diabetes-specific COVID-19 worries comprising 11 items, and general COVID-19 worries are rated on a scale of 1 (low) to 10 (high); section II is on sociodemographic and medical status in terms of diabetes type, diabetes complications, mental illness, glycemic control, symptoms of COVID-19, COVID-19 test status, and history of COVID-19-related hospitalization. Section III comprises social relations measures in isolation, loneliness, need for diabetes self-management, and diabetes distress. Section IV includes questions on changes in diabetes-related behaviors.

### Validation of the study instrument

The corresponding author adapted the available semi-structured questionnaire as per the regional sociocultural requirement. Only face validity of the questionnaire was assessed and all other investigators provided their inputs. The questionnaire was then finalized. The corresponding author is a consultant psychiatrist and trained the second author in administering the interview. The second author is a qualified and trained psychologist. The time taken for each interview was 20–30 min and the interviews were carried out in Odiya/Hindi as per the stated ease of the participant.

### Statistical analysis

Data were collected on Epicollect; analysis was carried out in Statistical Package for Social Sciences for Windows (SPSS Inc. Released 2018, version 25.0. Armonk, New York, USA). Kolmogorov–Smirnov tests were carried out to ascertain the normality of data. Between-group comparisons of continuous variables were made with independent *t*-test and two-way ANOVA; correlations were carried out with Pearson correlation test. We carried out all statistical analysis keeping statistical significance at 0.05.

### Sources of potential biases and measures taken

Consecutive patients’ telephone number was chosen from the clinic data to avoid any selection bias. Measurement bias was taken care of by random checking of the data by repeat telephonic calls to the respondents.

## RESULTS

**Figure 1:**
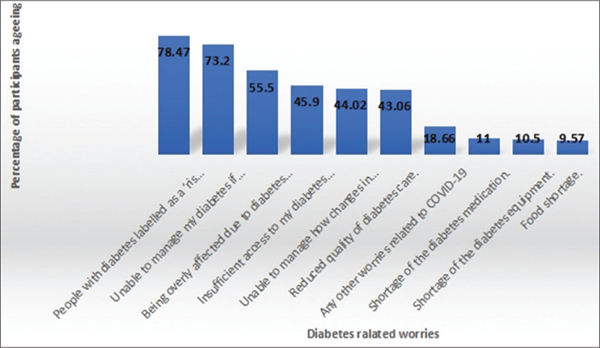
Participants reported diabetes-related worries.

We contacted 238 patients of which 225 agreed for a telephonic interview. However, the final cohort included the 209 patients who completed the entire interview. Of the 16 patients who could not complete the interview, the reasons were disconnection of telephonic conversation due to network issues, inability on the part of the participant to complete the interview due to competing priorities, unavailability of the patient, and phone number belonging to distant relatives which made data collection impossible. Of the 209 participants, 115 were males, 140 were in the age group 40–60, and 87 had attained college-level education. The detail sociodemographic characteristics are shown in Table 1. The mean score on a scale of 1–10 in COVID-19-related worries was 3.59 (standard deviation = 1.51]. About 80% of participants reported being worried about being at high risk for COVID-19. About three-fourths of participants reported feeling worried about managing diabetes if infected with diabetes, and about half said being overly affected by diabetes if infected with COVID-19. Details are shown in Figure 1. Younger age, being unemployed, and suffering from a mental disorder were significantly associated with COVID-19-related worries [Table 2]. Furthermore, poor glycemic control and presence of COVID-19 symptoms were associated with higher mean scores of COVID-19 worries. Three-quarters of the patient population reported feeling diabetes distress. Among the psychosocial variables, the experience of social isolation, diabetes distress and a shared sense of social support for managing diabetes showed a significant correlation with experience of COVID-19-related worries [Table 3].

## DISCUSSION

COVID-19 pandemic brought strict lockdown restrictions, which compounded the worries related to COVID-19 itself. Across the world, rates of depression and anxiety have soared.^[7]^ People with diabetes were at risk of contracting the virus and were facing difficulty in self-management. Medical consultation was redefined during the pandemic, and efforts were being made to provide quality health care to this population.^[8]^

Patients with diabetes are at risk to experience psychosocial problems. Diabetes-specific worries, distress and depression, are known to have bidirectional relation with diabetes. These psychosocial problems negatively impact how a patient manages himself/herself in managing diet, adhering to exercise routine, refilling prescriptions, routinely monitoring blood glucose levels, and going for regular medical consultations with the diabetologist.^[9]^ Diabetes distress is a negative emotional state specifically related to the diagnosis of diabetes, need for stringent self-management, everyday need for medications, and concerns about long-term complications of diabetes. Available literature suggests that about 44% of patients experience diabetes distress.^[10]^ The prevalence of diabetes distress reported by patients with diabetes during the COVID pandemic is nearly double the pre-COVID times.^[11]^

Among the sociodemographic variables, we found a young age experiencing more diabetes worries than more elderly age groups. Furthermore, being unemployed and suffering from a mental illness was strongly associated with more diabetes worries. Our findings are in tune with the reported experience in a diabetic population. Surveys worldwide also report a higher prevalence of worries in younger age and people with mental disorders because of their higher vulnerability. Social interactions are much higher in younger people than the old; lockdown restrictions reduced socialization opportunities and increased distress in the younger age group.^[7]^

Among the psychosocial variables, we also found feelings of social isolation and diabetes distress positively and significantly correlated with diabetes worries. Diabetes worries were negatively correlated with social support feeling. This correlation, though statistically significant, was of moderate size.

## CONCLUSIONS

People with diabetes experience more COVID-19-related worries. These worries are related to their diabetes care. Being young, unemployed, and having a mental illness is associated with experiencing COVID-19 worries. Social isolation and diabetes distress were also positively correlated with COVID-19 worries, whereas a lack of social support was associated with more worries. Poor glycemic control and presence of COVID-19 symptoms were also associated with higher COVID-19 worries. There is a need to provide psychosocial support to this at-risk population to improve their outcomes.

### Strengths, limitations, and generalizability

To the best of the authors’ knowledge, this is the only Indian study which has used a validated questionnaire for assessing the experience of COVID-19 worries in patients with diabetes mellitus. A single-center study and nonrandomization of the data limit the generalizability of the findings.

### Research quality and ethics statement

All authors of this manuscript declare that this scientific study is in compliance with standard reporting guidelines set forth by the EQUATOR Network. The authors ratify that this study required IEC review, and hence, prior approval was obtained IEC No. T/IM-NF/Psychi/20/100 dated August 26, 2020. We also declare that we did not plagiarize the contents of this manuscript and have performed a Plagiarism Check.

## Figures and Tables

**Table 1:** Study population sociodemographic and clinical characteristics

Variable	Descriptive statistics

Age	
18–40	27 (12.9)
41–60	140 (66.99)
>61	42 (20.09)
Gender	
Male	115 (55.02)
Female	94 (44.98)
Education	
Primary	49 (23.44)
Secondary	87 (41.63)
Tertiary	73 (34.93)
Employment status	
Employed and homemakers	147 (70.33)
Unemployed	62 (29.67)
Diabetes type	
Type 1	14 (6.69)
Type 2	195 (93.3)
Diabetes complications	
Retinopathy	20 (9.57)
Nephropathy	15 (7.18)
Neuropathy	5 (2.39)
Foot ulcers	14 (6.69)
Cardiovascular disease	8 (3.83)
COVID-19 symptoms	161 (77.03)
COVID-19 tested positive	
Self	30 (14.35)
Relatives	48 (22.96)
Hospitalized for COVID-19	
Self	25 (11.96)
Relatives	43 (20.57)

**Table 2:** Association of COVID-19 worries with sociodemographic and clinical variables

Variable	Category	*n* (%)	Mean	*P*

Gender	Male	115 (55.02)	3.57	0.47
	Female	94 (44.97)	3.62	
Age	18–40	26 (12.44)	4.54	<0.001
	41–60	140 (66.99)	3.61	
	>61	43 (20.57)	2.93	
Education	Primary	48 (22.97)	3.85	0.253
	Secondary	87 (41.63)	3.61	
	Tertiary	74 (35.41)	3.39	
Employment status	Employed	148 (70.81)	3.36	0.029
	Unemployed	61 (21.19)	3.68	
SES	Low	31 (14.83)	3.71	0.1
	Middle	54 (25.84)	3.41	
	Upper	29 (13.88)	3.1	
Type of DM	Type 1	13 (6.22)	3	0.147
	Type 2	196 (93.78)	3.63	
HbA1c (%)	<8	95 (45.45)	5.37 (1.43)	0.008
	8–9	52 (24.88)	4.94 (1.72)	
	>9	62 (29.66)	5.82 (1.33)	
Complications of diabetes	Present	124 (59.33)	3.55	0.64
	Absent	85 (40.66)	3.65	
Mental illness	Present	06 (2.87)	7.33	<0.001
	Absent	203 (97.12)	3.48	
COVID-19 symptoms	Present	161 (77.3)	5.73	0.03
	Absent	46 (22)	4.17	

SES: Socioeconomic status, DM: Diabetes mellitus, HbA1c: Glycated hemoglobin

**Table 3:** Correlation of COVID-19 worries with psychosocial variables

Psychosocial variables	COVID worries, *P* (*P*)

Isolation	0.401 (<0.001)***
Diabetes distress	0.441 (<0.001)***
DAWN total	−0.158 (0.023)**

** *P*<0.05 statistically significant

*** *P*<0.005 highly statistically significant. DAWN: Diabetes Attitudes, Wishes, and Needs
